# Synthesis, characterization, and application of reversible PDLLA-PEG-PDLLA copolymer thermogels *in vitro* and *in vivo*

**DOI:** 10.1038/srep19077

**Published:** 2016-01-11

**Authors:** Kun Shi, Ya-Li Wang, Ying Qu, Jin-Feng Liao, Bing-Yang Chu, Hua-Ping Zhang, Feng Luo, Zhi-Yong Qian

**Affiliations:** 1 State Key Laboratory of Biotherapy and Cancer Center, West China Hospital, Sichuan University, and Collaborative Innovation Center for Biotherapy, Chengdu, 610041, China

## Abstract

In this study, a series of injectable thermoreversible and thermogelling PDLLA-PEG-PDLLA copolymers were developed and a systematic evaluation of the thermogelling system both *in vitro* and *in vivo* was performed. The aqueous PDLLA-PEG-PDLLA solutions above a critical gel concentration could transform into hydrogel spontaneously within 2 minutes around the body temperature *in vitro* or *in vivo*. Modulating the molecular weight, block length and polymer concentration could adjust the sol-gel transition behavior and the mechanical properties of the hydrogels. The gelation was thermally reversible due to the physical interaction of copolymer micelles and no crystallization formed during the gelation. Little cytotoxicity and hemolysis of this polymer was found, and the inflammatory response after injecting the hydrogel to small-animal was acceptable. *In vitro* and *in vivo* degradation experiments illustrated that the physical hydrogel could retain its integrity as long as several weeks and eventually be degraded by hydrolysis. A rat model of sidewall defect-bowel abrasion was employed, and a significant reduction of post-operative adhesion has been found in the group of PDLLA-PEG-PDLLA hydrogel-treated, compared with untreated control group and commercial hyaluronic acid (HA) anti-adhesion hydrogel group. As such, this PDLLA-PEG-PDLLA hydrogel might be a promising candidate of injectable biomaterial for medical applications.

Temperature-sensitive polymeric hydrogels have been extensively investigated as promising biomaterials for sustained drug delivery, cell encapsulation, tissue regeneration and post-operative adhesion prevention^1^,^2^,^3^. Typically, the polymer aqueous solutions are sol (solution) state at room temperature or lower, but spontaneously turn into non-flowing gels after administration in response to the physiological temperature. Such unique properties enable pharmaceutical agents or cells to be easily incorporated into the polymer aqueous solutions by simply mixing in the sol state, followed by injecting the corresponding formulations into a target tissue to form a standing gel *in situ*, acting as a controlled drug delivery depot or scaffold materials. Meanwhile, such a chemical-reaction free approach with minimal invasiveness is very beneficial for medical applications^4^,^5^.

Commercially available triblock copolymers composed of poly(ethylene glycol-propylene glycol- ethylene glycol) (Pluronics or Poloxamers) exhibit a temperature-induced reversible sol–gel transition and have been reported for the sustained delivery of several drugs^4^,^6^. Unfortunately, poloxamer are non-biodegradable, potentially toxic and eroded rapidly at the injection after administration, which limit their utility in biomedical applications to a certain extent^7^,^8^. Therefore, block copolymers consisted of poly(ethylene glycol) (PEG) and biodegradable polyesters, such as poly(lactic acid) (PLA)^9^,^10^, poly(lactic acid-co-glycolic acid) (PLGA)^11^,^12^, poly(caprolactone) (PCL)^13^,^14^, and poly(caprolactone-co-lactic acid) (PCLA)^3^,^15^ have been developed to obtain biodegradable and biocompatible thermogelling polymers, and impressive progress have been made over the past decades.

In particular, thermogelling copolymers based on PEG/PLA have received remarkable attention since the PEG-PLLA-PEG copolymers were developed by Jeong and coworkers as the first biodegradation and thermosensitive hydrogel^9^. The PEG-PLLA-PEG triblock copolymers were synthesized in two steps by coupling the firstly prepared MPEG-PLLA diblock polymers using hexamethylene diisocyanate (HMDI) as coupling agent. Aqueous solution of the triblock copolymers underwent a gel-sol transition as temperature increased and the *in vitro* sustained release of dextran from this hydrogel was investigated. Later on, star-shaped PLLA-PEG^16^ and PEG-PDLLA-PEG triblock^17^ copolymers were found to possess the similar gel-sol transiton. But the gel-sol transition property of these copolymers mentioned above may not suitable for encapsulation of protein or some drugs at higher temperature and injection of the hydrogel at elevated temperature is uncomfortable for patients^5^. Hence, many attempts were taken to find PEG/PLA-based hydrogels with lower critical solution temperature (LCST) around body temperature. Hydrogels formed from the stereocomplexation of poly(L-lactide) (PLLA) and poly(D-lactide) (PDLA) blocks were reported to show an expected sol-gel transition as temperature increased^18^. Although the stereocomplex-induced gelation of the enantiomeric copolymers could be realized through the mixing of enantiomeric triblok copolymers^19^,^20^, star-shaped copolymers^21^, or triblok and star-shaped copolymers^22^, the gelation was irreversible and depended on a restricted range of polymer composition. Besides, multiblock PEG/PLLA^23^ and PLA-PEG-PLA triblock stereo-copolymers with varying L-/DL-LA ratios^24^ were developed to exhibit a desired sol-gel-sol transition upon heating. The effects of block length, composition of copolymers and additives on the phase transition behavior were discussed as well.

However, to the best of our knowledge, there is no report on the PDLLA-PEG-PDLLA triblock copolymers showing reversible temperature-sensitive gelation so far. Herein, we developed a novel injectable thermogelling PDLLA-PEG-PDLLA triblock copolymer hydrogel by modulating the composition and block length of the copolymers, which exhibiting reversible and sharp sol-gel transition between ambient temperature and body temperature. The PDLLA-PEG-PDLLA copolymers were synthesized in one step without using any possibly toxic coupling agent, resulting in a facile and safe approach. In this paper, the structure–property relationship of the reversible sol–gel transition, gelation kinetics and mechanical properties were studied in detail. Furthermore, we will present a systematic study of the thermogelling PDLLA-PEG-PDLLA copolymers both *in vitro* and *in vivo*, including cytotoxicity, biocompatibility and dynamic biodegradation investigation. Compared to plenty of investigations of biodegradable medical materials, the systematic evaluation of the biocompatibility and degradation profiles of injectable synthetic hydrogels, especially of thermogelling PEG/PLA copolymers, is very limited^5^,^23^,^25^. The present study might be meaningful for the thermogelling PEG/PLA copolymers in biomedical and biopharmaceutical applications. In our previous work, we have also investigated a thermoreversible physical hydrogel based on PCL-PEG-PCL (PCEC)^26^, which have shown great potential in drug delivery and post-operative abdominal adhesion prevention^27^,^28^. But the solution of the PCEC tended to be unstable and gel at room temperature due to the crystallization of the polyesters blocks, and this can affect syringeability. From a practical point of view, the PDLLA-PEG-PDLLA copolymer might be more convenient and has broader application prospect in contrast, because it showed pronounced sol phase stability at room temperatures and could be used as an injectable *in-situ* formed thermogel. Finally, the application in post-operative anti-adhesion of the PDLLA-PEG-PDLLA hydrogel system was evaluated in a rat model of sidewall defect-cecum abrasion as well.

## Results and Discussion

### Synthesis and characterization of PDLLA-PEG-PDLLA triblock copolymers

The triblock copolymers were synthesized via ring-opening polymerization of D,L-lactide using PEG as initiator and stannous octoate as catalyst. A series of PDLLA-PEG-PDLLA copolymers with different molecular weight and block ratio were synthesized in this work to optimize the hydrogel formulation and mechanical properties. In this paper, the copolymers were denoted as L_B_–E_A_–L_B_ (PLEL), where A and B represent the theroretical number average molecular weights (Mn) of PEG and PDLLA blocks respectively. ^1^H-NMR analysis (see Supplementary Fig. S1) and GPC measurements were performed to characterize the chemical structure of the obtained copolymers, which were summarized in Table 1. All of these results indicated the successful synthesis of PDLLA-PEG-PDLLA triblock copolymers designed by controlling the feed composition and the yield were more than 90%.

The synthesized PDLLA-PEG-PDLLA triblock copolymers consisting of hydrophilic PEG block and hydrophobic PDLLA block exhibited amphipathicity in aqueous solution. The block length, PEG/polyester ratio and molecular weight played important role in the dissolution of the amphiphilic block copolymers^29^. Among these copolymers prepared, copolymers (S1, S2, S3 and S6) with PEG/PDLLA ratio about 0.5 could dissolve in water easily, while L_1700_–E_1500_–L_1700_ (copolymer S4) having a smaller PEG/PDLLA ratio was hard to dissolve. Although L_2000_–E_2000_–L_2000_ (copolymer S5) possessed an appropriate PEG/PDLLA ratio of 0.5, its long block of hydrophobic PDLLA gave rise to strong hydrophobility in water. Among these soluble copolymers, copolymers (S1 ~ S3) underwent an apparent sol-gel transition, whereas L_1000_–E_2000_–L_1000_ (copolymer S6) solution didn’t show sol-gel transition in the temperature range of 10 ~ 60 °C due to its too long block of hydrophilic PEG. PCEC (1000-1000-1000) exhibited sol-gel transition as described in our previous reported paper^13^,^26^. These phenomena indicated that the thermogelation of such an amphiphilic polyester-polyether block copolymers system is attributed to the delicate balance between hydrophobicity of the polyester block and hydrophilicity of the PEG segment. Notably particularly, the dissolution of the obtained PDLLA-PEG-PDLLA copolymers could be done by stirring at low temperature, whereas the preparation of PCEC polymer aqueous solution needed a cumbersome heating and quenching cycle procedure^14^,^23^.

### Micellization and the proposed hierarchical structure of the gel

**Figure 1:**
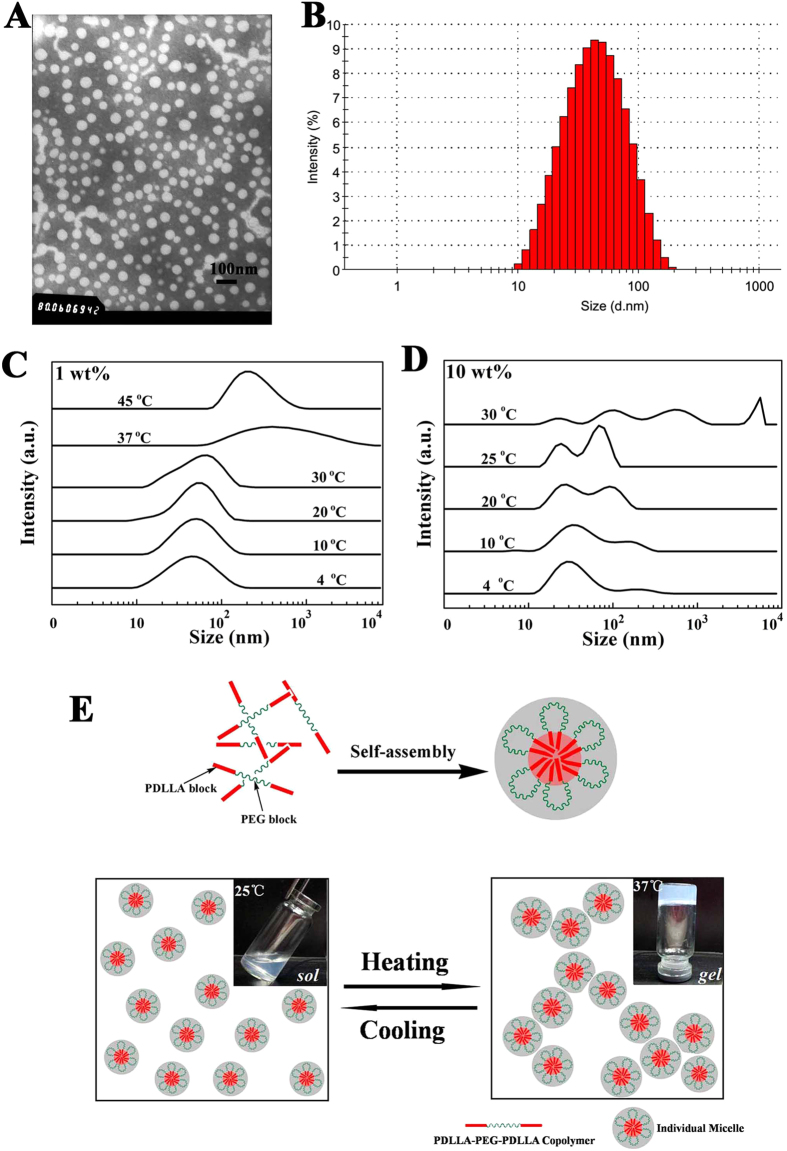
Characterization of PDLLA-PEG-PDLLA micelle at low concentrations and schematic diagram showing the thermogelling behavior. (**A,B**) TEM image and particle size of micelles at 25 °C (S3, 0.1 wt%). (**C,D**) Particles size distribution spectrum of micelles (S3, 1 wt% and 10 wt%) at different temperatures, each measurement performed after an equilibration for 10 min. (**E**) The schematic diagram of the process of temperature-induced physical gelation. The amphiphilic block copolymers self-assemble into core-shell-like micelles in aqueous solution (25 wt%) and then gelate between room temperature and body temperature due to the augment and aggregation of the micelles after heating.

A better understanding of temperature-dependent sol-gel transition mechanism is important for optimizing properties of the hydrogel. Usually, amphiphilic polyester-polyether block copolymers could self-assemble into core-shell-like micelles in water and the micellar aggregation was thought to be involved in the underlying mechanism of physical sol-gel transition^12^,^24^. Therefore, we initially investigated the self-assembled PDLLA-PEG-PDLLA copolymer micelles in different conditions. Transmission electronic microscopy (TEM) observation and dynamic light scattering (DLS) measurement certified that the micelles were dispersed as individual spherical nanoparticles with 40 ~ 50 nm particle size at a low concentration (0.1 wt%) (Fig. 1A,B). Aggregation of the micelles at higher concentration was further detected by DLS under various temperatures to study the proposed hierarchical structure during the gelation. Micelles in the 1.0 wt% polymer solution exhibited unimodal size distributions and the size of micelles increased slightly with the rising of temperature from 4 °C to 30 °C, followed by an abrupt augmentation around 37 °C, which may enable more significant aggregation at higher concentrations (Fig. 1C). As expected, multimodal size distributions and an obvious aggregating behavior were observed gradually over 4 ~ 30 °C when micelles were in the 10 wt% polymer solution, corresponding to the sol-gel transition (Fig. 1D). Furthermore, these results also suggested that the sol-gel transition affected not only by the temperature but also by the concentration of the copolymer solution.

Although the underlying mechanism of the physical gelation of amphiphilic block copolymers in water remains as a challenging fundamental problem in this field, all of our measurements so far support the two-step process of temperature-induced physical gelation^23^,^30^, which may be schematically illustrated as in Fig. 1E. In aqueous solution, the hydrophobic PDLLA in the copolymer constitutes the self-assembled micelle’s core due to hydrophobic interaction, and the hydrated PEG blocks forms the hydrophilic shell. At low temperature, the small micelles flow freely and the aqueous solution seems to be a sol-like suspension. With increase in temperature, the micelle size augments followed by the aggregation and packing between micelles, resulting in a physically cross-linked non-flowing gel structure around body temperature.

### Sol-gel transition of PDLLA-PEG-PDLLA hydrogel

**Figure 2:**
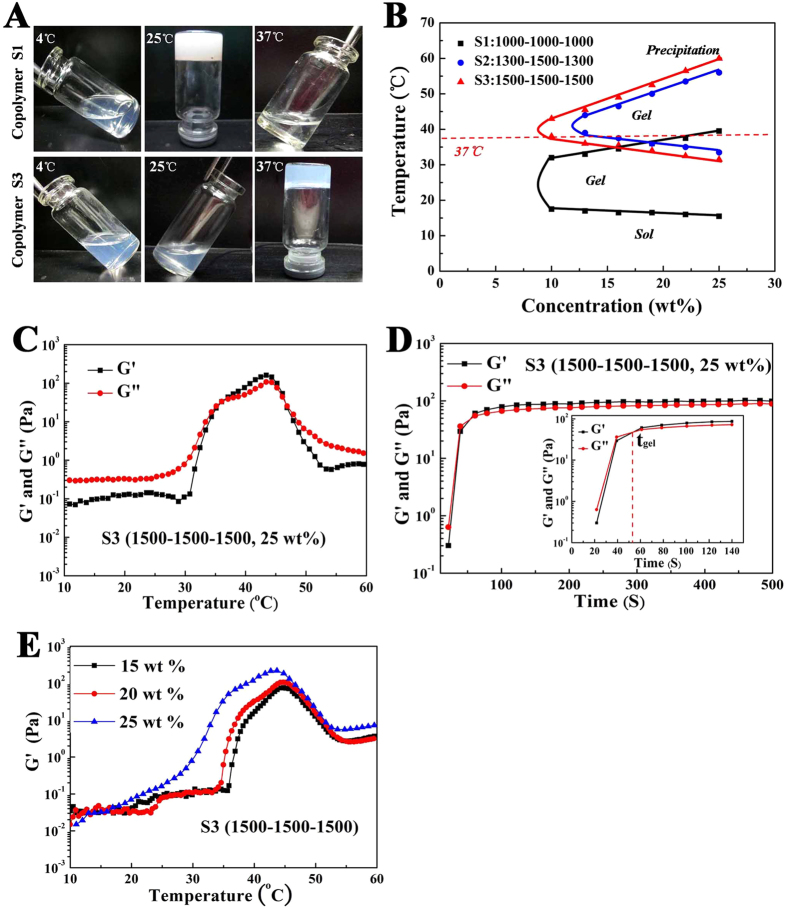
The thermogelling behavior assay and rheological analysis of the obtained. PDLLA-PEG-PDLLA hydrogel. (**A**) Photographs of the copolymer solutions at different temperatures. Copolymer L_1000_–E_1000_–L_1000_ (S1, 25 wt%) gelates between 4 °C and room temperature, and precipitated around 37 °C. Copolymer L_1500_–E_1500_–L_1500_ (S3, 25 wt%) exhibited a sol at 4 °C and room temperature, and gelated around body temperature. (**B**) Sol–gel phase transition diagram of the hydrogel tested by the tube-inverting method. (**C**) Temperature-dependence of storage (G’) and loss modulus (G”) for the copolymer aqueous solution (S3, 25 wt%) as a function of temperature. (**D**) Gelation times of the copolymer solutions (S3, 25 wt%) at 37 °C. (**E**) Change in G’ of the copolymer solutions at different concentrations (S3, 15 wt%, 20 wt%, 25 wt%) as a function of temperature.

The phase transition of the PDLLA-PEG-PDLLA copolymers aqueous solutions was investigated by test-tube-inversion method and dynamic rheological analysis. Figure 2 (A,B) presented the phase transition diagram of PDLLA-PEG-PDLLA copolymers aqueous solutions upon temperature and concentration obtained via the tube-inverting method. Above a critical gel concentration (CGC), the phase transition process consisted of sol, gel and precipitation three basic physical states upon heating, leading to a lower critical gelation temperature (LCGT) from sol-gel and an upper critical gelation temperature (UCGT) from precipitation. According to the phase transition diagram, the LCGT and UCGT changed with the variation of concentration. With increasing polymer concentration, the gelation taken place at lower temperature, whereas the precipitation occurred at higher temperature, as a result of higher micelle concentrations and strengthen aggregation between micelles. When the PEG/PDLLA ratio was kept constant (1/2), augment in total molecular weight from 3,000 for S1 to 4,500 for S3, both LCGT and UCGT increased significantly, while the overall curve shape remained almost unchanged, resulting in the shift of the gel window to higher temperature. In addition, at a given PEG block (1,500), the increase in the length of PDLLA block made decrease in CGC and LCGT, but increase in UCGT. In other words, the gel range in the phase diagram becomes larger with the increase of the PLA blocks when the PEG block was kept constant.

It is obvious that the phase transition behavior was significantly altered depending on the molecule weight, block length and polymer concentration. Thus, we could adjust the gelation temperature of the hydrogel in a physiologically important temperature range by modulating these factors. An appropriate LCGT between room temperature and body temperature means a realizable injection at room temperature and rapid gelation in site during operation, while a higher UCGT above 50 °C implies a stable gel phase after applying the hydrogel *in vivo*. Copolymer L_1000_–E_1000_–L_1000_ (S1) gelled spontaneously under the room temperature and precipitated around 37 °C (Fig. 2A,B), which was undesirable for practical application. Copolymer L_1300_–E_1500_–L_1300_ (S2) had a narrower gelation window when compared with L_1500_–E_1500_–L_1500_ (S3) (Fig. 2B). Overall, copolymer L_1500_–E_1500_–L_1500_ (S3) exhibited the optimized gelation temperature (Fig. 2A,B), supporting the choice of L_1500_–E_1500_–L_1500_ (S3) in formulations chosen for further studies.

Accompanying with the thermogelling behavior, the concentrated polymer solution underwent a significant change in mechanical properties. Dynamic rheological measurements were carried out to quantitatively observe the sol-gel transition of L_1500_–E_1500_–L_1500_ (S3) solution (Fig. 2C–E). The close value of storage modulus (G’) and loss modulus (G”) in the gel phase of current polymers indicates the semisolid nature of the gel. Thus, the sol-gel transition was defined as the point at which G’ increased larger than G”^23^. Both G’ and G” of L_1500_–E_1500_–L_1500_ solution (S3, 25 wt%) were very low (less than 1 Pa, G’ < G”) and were essentially independent of temperature from 4 °C to 30 °C, which further proved the injectability of this copolymer solution without the risk of syringe clogging upon injection. As temperature increased around body temperature, both G’ and G” raised abruptly more than 3 magnitudes, corresponding to the sol-gel transition (Fig. 2C). The differential growth rates between G’ and G” leaded to the growth of the intensity of the hydrogel. Gelation time of the copolymer solutions at 37 °C was investigated as well, just as Fig. 2D shown. The physical hydrogel formed within about 50 ~ 60 s at 37 °C, and this gelation time is quite good for the applications of *in-situ* gel-forming hydrogels, especially for tissue engineering operation. We also fund that, with the increase of copolymer concentration, storage modulus (G’) enhanced accordingly, which may improve shape persistence after injecting *in vivo* (Fig. 2E).

### Thermally reversible gelation of PDLLA-PEG-PDLLA hydrogel

**Figure 3:**
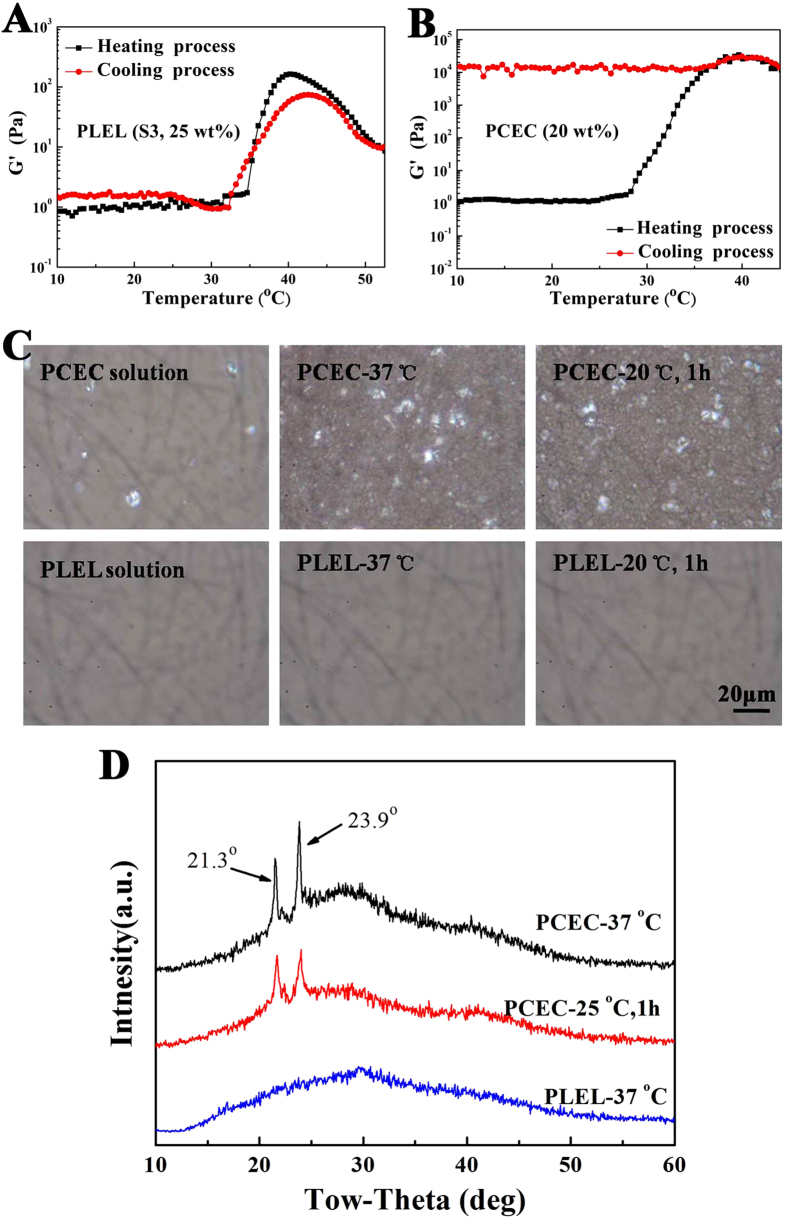
The thermally reversible gelation study of the PDLLA-PEG-PDLLA (PLEL) hydrogel (S3, 25 wt%) comparing with the PCL-PEG-PCL (PCEC) hydrogel (20 wt%). (**A,B**) Change in G’ of the PLEL copolymer solution and PCEC copolymer solution under heating and cooling process. (**C**) Polarized optical microscopic images of the PLEL copolymer solution and PCEC copolymer solution under different conditions: images of the polymer solutions at 20 °C; images of the polymer hydrogels formed by direct heating at 37 °C; images of the samples after 1 hour at 20 °C. The scale bar was 20 μm. (**D**) X-ray diffraction pattern of the PLEL hydrogel formed instantaneously at 37 °C and PCEC hydrogel formed at 20 °C for 1 hour or formed instantaneously at 37 ^o^C.

It was worth mentioning that, the sol-gel transition of the PDLLA-PEG-PDLLA hydrogel was thermally reversible. The non-flowing gel formed by increasing the temperature of the polymer aqueous solution became a sol again when cooled to low temperature. No evidence of change has been observed yet during the numerous repeats of the interconversion between sol and gel state. The thermally reversibility of the PDLLA-PEG-PDLLA (PLEL) hydrogel was further proved by the rheological measurement via testing the G’ under heating and cooling process, just as Fig. 3A shown. Although slight hysteresis between the heating and cooling curves was found, the transition temperature and the variation trend of G’ were almost similar, illustrating the thermoreversibility of the physical gelation. Interestingly, the thermally reversibility of the PDLLA-PEG-PDLLA polymer hydrogel was really different from the physical gelation of our previously reported PCL-PEG-PCL (PCEC) thermosensitive hydrogel (Fig. 3B), which might result from different gelation mechanisms.

To understand this phenomenon, polarized optical microscopic tests of the PDLLA-PEG-PDLLA (PLEL) copolymer solution (25 wt%) and PCL-PEG-PCL (PCEC) copolymer solution (20 wt%) were carried out to study the morphology during the gelation (Fig. 3C). Few crystalline phase was observed in the photograph of the PCEC copolymer aqueous solution taken after dropping the polymer solution on the slide glass at 20 °C. When the PCEC sample was heaing to 37 °C, an opaque gel formed instantaneously and the polarized optical microscopic image showed a bundle of the crystalline morphology. The same opaque gel and significant crystalline phase were also observed in the image of PCEC sample taken after 1 hour at 20 °C. Differently, the polarized optical microscopic image of the PDLLA-PEG-PDLLA copolymers aqueous solutions was the same over the same procedure and no crystalline morphology was found neither in sol state at 20 °C nor in gel state at 37 °C.

Simultaneously, the X-ray diffraction of the turbid PCEC hydrogel formed at 20 °C for 1 hour or formed instantaneously at 37 °C showed strong diffraction peaks at 21.3^o^ and 23.9^o^ corresponding to crystalline PCL, whereas the PLEL hydrogel did not present any diffraction peak (Fig. 3D). All of these results suggested that the crystallization of the PCL block is involved in the gelation of the PCEC copolymer aqueous solution both in the low-temperature gel and the thermogel, which is consistent with Jeong’s work reported previously^14^. On the contrary, the thermogelling of the PDLLA-PEG-PDLLA copolymers aqueous solution arised from the physical interaction of the copolymer micelles with no crystallization, leading to the thermally reversible gelation. Therefore, these also demonstrated that the composition of the polyester played an important role in the gelation behavior in addition to the block length and polymer concentration.

### Biocompatibility of PDLLA-PEG-PDLLA hydrogel *in vitro* and *in vivo*

**Figure 4:**
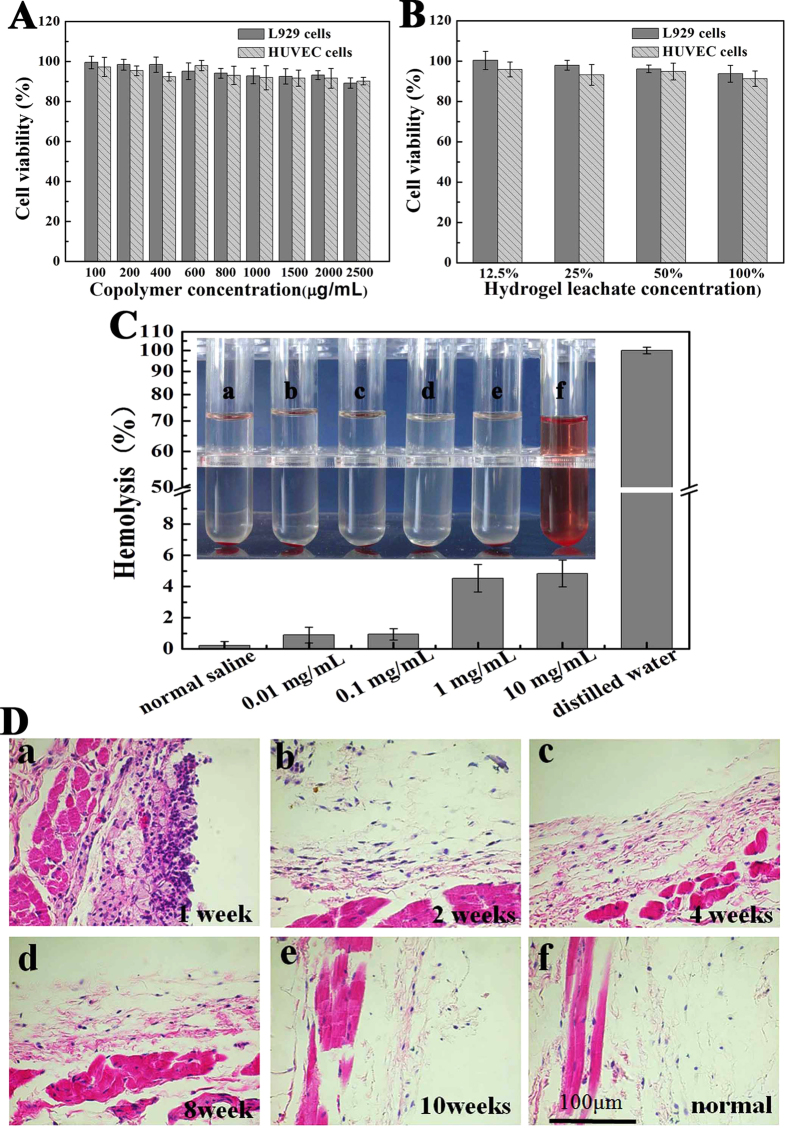
Biocompatibility of PDLLA-PEG-PDLLA hydrogel (S3, 25 wt%) *in vitro* and *in vivo*. (**A,B**) Effect of the copolymer and hydrogel extract on cell viability of L929 and HUVEC cells measured by MTT assay. Data were presented as mean ± SD (n = 5). (**C**) Hemolytic test of PDLLA-PEG-PDLLA triblock copolymer (S3). This photo was taken after 3 hours’ reaction. Sample (f) was distilled water used as positive control, while sample (a) was normal saline used as negative control. The concentration of PDLLA-PEG-PDLLA copolymer was 0.01 mg/ml (b), 0.1 mg/ml (c) 1.0 mg/ml (d), and10.0 mg/ml (e). Hemolysis rate in each group were presented as mean ± SD (n = 3). (**D**) HE-staining of surrounding tissues at designated time (a to e) after dorsal subcutaneous injection of PDLLA-PEG-PDLLA solution (S3, 25 wt% in PBS, PH = 7.4) into BALB/c mice for examination of the inflammatory response. The normal tissue taken as the blank control was shown in (f). Magnification: 400 ×. The images was representative of n = 3.

The cytotoxicity of PDLLA-PEG-PDLLA copolymer and hydrogel extract was evaluated by cell viability assay using L929 and HUVEC cells. Fibroblasts and vascular endothelial cells play extremely important roles during the process of wound healing^31^,^32^. Therefore, the biocompatibility of PDLLA-PEG-PDLLA material with these cells is directly related to the tissue regeneration applications. According to Fig. 4A, both L929 and HUVEC cells viability slowly decreased with increase of the copolymer concentration. But even at a high concentration of 2.5 mg/ml, 85% higher of cell viability was detected yet. The viability of L929 and HUVEC cells cultured with hydrogel extract was also approximately 90% (Fig. 4B). These results indicated that PDLLA-PEG-PDLLA copolymer and hydrogel extract had minimal cell cytotoxicity and were safe materials.

In view of the fact that the copolymer hydrogel should cover the injury site when it used in tissue regeneration application, so we also observed the hemocompatibility of the copolymer *in vitro*. A hemolysis test was used, following the protocol of ISO 10993 as an international standard for the biological evaluation of medical devices. The standard of no hematotoxicity usually refers to a hemolysis percentage below 5%^33^. As the Fig. 4C shown, the synthesized PDLLA-PEG-PDLLA copolymer at concentrations ranging from 0.01 to 10 mg/ml exhibited little hemolysis percentage less than 5%, which was similar to the negative control (the normal saline solution). Compared with the positive control (distilled water), all of our polymer solutions revealed significant difference (P < 0.05), while the positive control was set as 100% of hemolysis. Therefore, the hemolysis capacity of PDLLA-PEG-PDLLA copolymer was negligible.

The immunological response of the BALB/c mice to the PDLLA-PEG-PDLLA copolymer hydrogel after dorsal subcutaneous injection was investigated by observing the connective and muscular tissues surrounded the gel at multiple time points (Fig. 4D). At the first week, the surrounding injected site had a thicker infiltrate densely populated by neutrophils, lymphocytes and macrophages, characteristic of acute inflammation^34^. Subsequently, the number of the neutrophils, lymphocytes and macrophages reduced gradually during 2 ~ 6 weeks, suggesting that the acute inflammatory reaction was gradually replaced by a mild chronic inflammatory^25^. The PDLLA-PEG-PDLLA implants had an acute and chronic inflammatory response, which lasted for more than 6 weeks. Ten weeks later, the tissue sample from the injection site was almost recuperated to the normal tissue (the implant had been absorbed by this time). Neither significant muscle damage, nor tissue necrosis, hyperemia, edema, hemorrhaging were observed throughout the experiments ((see Supplementary Fig. S2)). In sum, the PDLLA-PEG-PDLLA hydrogel might have acceptable biocompatibility for biomedical applications.

### *In vitro* degradation of PDLLA-PEG-PDLLA hydrogel

**Figure 5:**
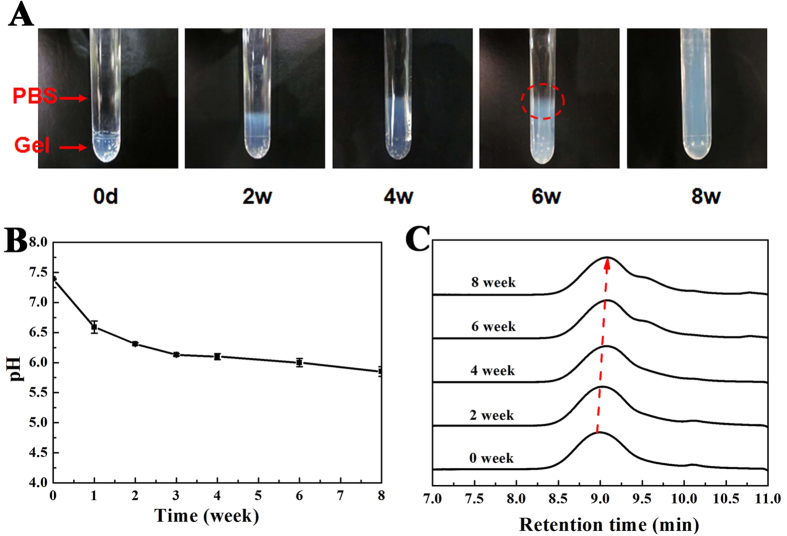
*In vitro* degradation of the PDLLA-PEG-PDLLA hydrogels (S3, 25 wt%) in PBS (initial pH 7.4) at 37 °C. (**A**) Optical images of the hydrogels at the indicated degradation time and the images was representative of n = 3 at each time. The PBS level was highly above the upper edge of the hydrogel and thus beyond the display field in these images. The water-soluble product diffused out of the gel was emphasized by the dashed ellipse in the image (6 weeks); (**B**) The change of the medium pH in degradation of hydrogels; (**C**) GPC profiles of PDLLA-PEG-PDLLA copolymer after the indicated degradation period *in vitro*, the dashed arrow indicated the decrease of peak molecular weight.

The *in vitro* degradation behavior of PDLLA-PEG-PDLLA hydrogel was evaluated under the mimetic physiological condition (37 °C, pH 7.4). Figure 5A displayed the gross views of the remaining hydrogels (S3, 25 wt%) at predetermined degradation time points. Equilibrating with the buffer system, the samples gradually swelled nearly 100% and the surface was eroded within the initial 4 weeks. At about the sixth week, a significant amount of water-soluble products were dissolved into the buffer solution and it was difficult to retain the gel shape (see the dashed ellipse in Fig. 5A (6 weeks)), resulting in the disintegration of hydrogel. Finally, the gel in the tube became free-flowing liquids after incubation for 8 weeks. The pH change of the buffer medium was also checked during degradation with the results shown in Fig. 5B. The pH of buffer was observed to decrease slowly as a result of the generation of D,L-lactide and low-molecular-weight oligomer even if the medium was replaced every 4 days. Nevertheless, the pH was still among 6.0 ~ 7.4 for five weeks, indicating a mild acidic effect of the hydrogel during degradation, which might be attributed to the high water content in hydrogel and the rapid diffusion of acidic degradation products out of gel. In addition, the remaining hydrogels were collected and measured by GPC at each time point. As shown in Fig. 5C, the single peak profile almost maintained in the examined periods of degradation, but the retention time at peak maximum slowly shifted to larger values and the chromatograms were gradually broadened as a function of degradable time, which reflected a steady decease of MW as the hydrolysis proceeded. Our research revealed that degradation of the PDLLA-PEG-PDLLA hydrogels proceeded by steady hydrolysis of ester bonds followed by the erosion of gel in water.

### *In vivo* gel formation and degradation study of PDLLA-PEG-PDLLA hydrogel

**Figure 6:**
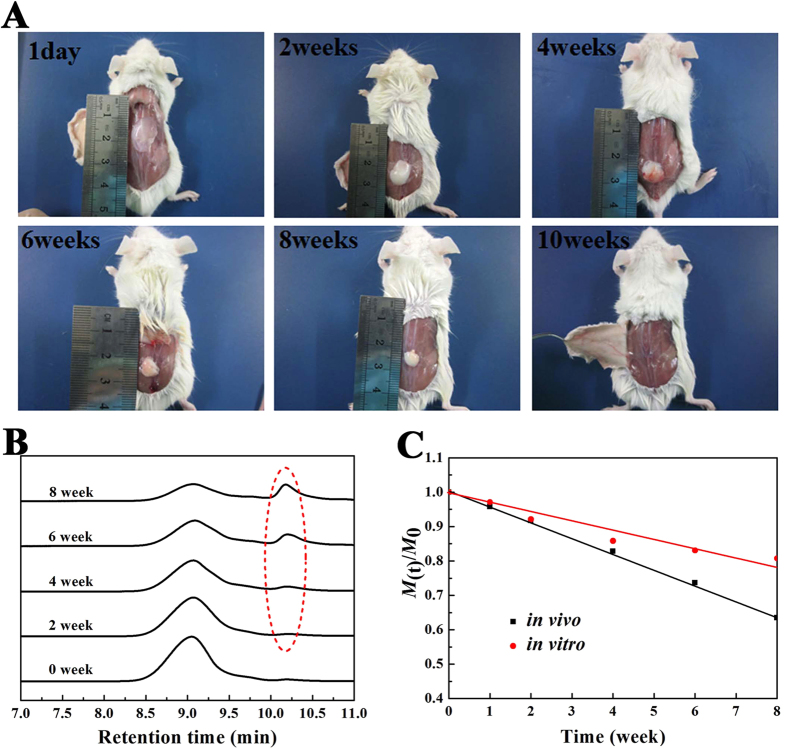
*In vivo* degradation of the hydrogels after subcutaneous injection of 25 wt% copolymer in the normal saline solution into the back of the BALB/c mice. (**A**) *in vivo* gel maintenance. Images were taken at the pre-determined time after subcutaneous administration. The images was representative of n = 3 at each pre-determined time. The remaining hydrogel became smaller with time and completely disappeared by week 10; (**B**) GPC traces of PDLLA-PEG-PDLLA copolymer collected during *in vivo* degradation after subcutaneously injected in mice. The dashed circle emphasized the peaks of degradation products of low molecular weights; (**C**) Change of normalized molecular weight (M_(t)_/M_0_) of copolymers in the remaining hydrogels during *in vitro* and *in vivo* degradation. Here, M_0_ represented the MW of the copolymer before degradation.

*In vivo* gel formation and maintenance were observed and confirmed in BALB/c mice by dorsal subcutaneous injection with a syringe needle (25-gauge) at room temperature. The injected copolymer solution (S3, 25 wt%) formed a round or irregularly shaped protrusion rapidly after subcutaneous injected into the mice backs. Figure 6A demonstrated some typical pictures taken on the 1st day, second week, fourth week, sixth week, eighth week and tenth week after subcutaneous administration. On visual observation, the hydrogel maintained its volumetric integrity for several weeks and became smaller upon time, with the obvious reduction of size at 6 weeks and complete disappearence at about the tenth week. In addition, the intensity of the remaining hydrogel decreased with time. The biodegradable PDLLA-PEG-PDLLA hydrogel with rapid *in-situ* gel formation and long gel persistence *in vivo* implies a promising application for longer-lasting drug delivery.

Moreover, the remaining gels at each time point were collected and the MWs of the recovered copolymers were detected by GPC to detect the degradation process. Figure 6B presented the time-dependent profiles of MWs of PDLLA-PEG-PDLLA hydrogels during *in vivo* degradation. A steady decrease of MW was illustrated by an increase of the retention time as the hydrolysis went on. Different from *in vitro* degradation, the multimodal, rather than the unimodal, were observed gradually during the *in vivo* degradation (see the dashed circle d in Fig. 6B), which might due to the slowly elimination of degraded fragments of low MW in the subcutaneous layer of the mice. For convenience of analysis, the change of molecular weight (M(t)) during both *in vitro* and *in vivo* degradation are presented in Fig. 6C, and the decreases of MW were roughly linearly fitted. The comparison indicates that the *in vivo* degradation is faster than *in vitro* degradation. The difference may be caused by some enzyme-assisted degradation *in vivo*, as was described in a similar biodegradable polyester-polyether hydrogel^3^.

### *In vivo* application and evaluation of peritoneal adhesion prevention efficacy in rats

**Figure 7:**
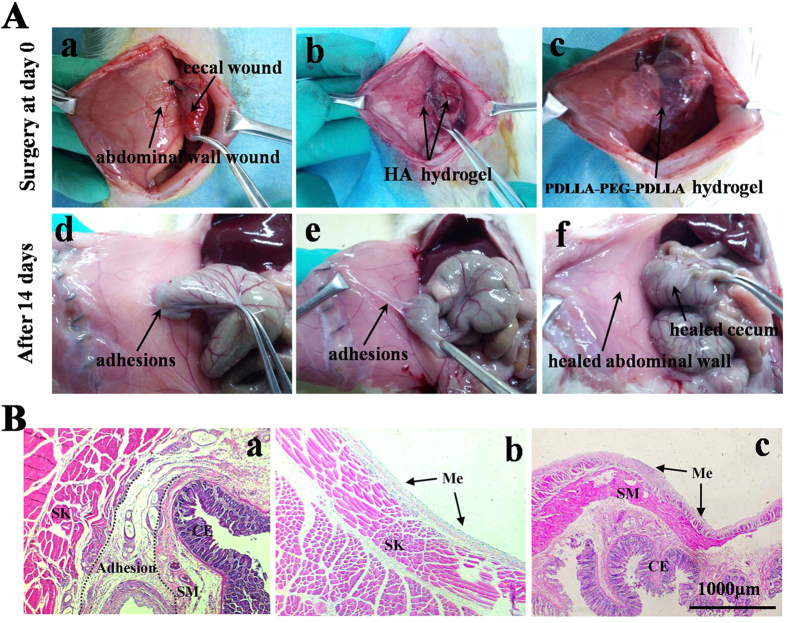
*In vivo* application and evaluation of adhesion-prevention effect in rat model. The images was representative of n = 8. (**A**) Photographs of animal experiments of post-operative adhesions. (a to c) In operation; (d to f) gross observations of the efficacy of prevention adhesion after 14 days; (a and d) The untreated defect was used as the negative control group and a firm adhesion was observed 14 days after operation; (b and e) HA hydrogel applied on the injury sites and moderate adhesion was observed in (d); (c and f) The injury sites were treated by PDLLA-PEG-PDLLA hydrogel and no apparent adhesion was observed in (f), with healed abdominal wall and cecum. (**B**) Optical micrographs of HE-straining slices for the injured site 14 days after surgery. (a) Adhesion between the defective cecum and abdominal wall from the animal without treatment. (b and c) Healed abdominal wall (b) and cecum (c) treated with the PDLLA-PEG-PDLLA hydrogel. A thin layer of remesothelialized tissue containing a larger number of mesothelial cells formed on the defect site. ME: mesothelial cells; SK: abdominal wall skeletal muscle; SM: visceral smooth muscle; CE: cecal mucosa. Magnification: 50 ×.

Post-operative peritoneal adhesions are inevitable consequences of abdominal or pelvic surgery and may cause severe complications^35^,^36^. Among the numerous strategies adopted to prevent post-operative adhesions, physical barrier systems are accepted as the most effective approach. Especially, biodegradable thermosensitive hydrogels have gained increasing attention serving as a post-operative adhesion barrier materia^13^,^37^. Herein, the adhesion prevention efficacy of the *in situ* formed PDLLA-PEG-PDLLA hydrogel was evaluated using a rat model of sidewall defect and bowel abrasion, as demonstrated in (Fig. 7A(a)). During the operation, the increased temperature in the abdominal cavity promoted the formation of PDLLA-PEG-PDLLA hydrogel (S3, 25 wt%) within 2min, conforming to the shape of the applied defects (Fig. 7A(b)). The transparent commercial HA anti-adhesion formulation rapidly diffused and formed a thin layer covering the wound and surrounding tissues after being injected to the defects (Fig. 7A(c)). All of the rats were sacrificed and dissected two weeks after the surgery to assess the status of adhesions. Some of the typical photos were presented in Fig. 7A(d–f), and the statistical analysis of the adhesive events was shown in Table 2. On gross examination, all rats in the untreated control group (n = 8) suffered from score 3 adhesions, the injured abdominal wall firmly adhered to the cecum, and the conglutinations could only be separated by cutting (Fig. 7A(d)). Although the formation of adhesion was reduced in the HA anti-adhesion hydrogel treated group, most of the animals still developed score 1 to 3 adhesions (Fig. 7A(e)). The disappointment performance of the HA anti-adhesion hydrogel may due to its short maintenance on the defects and rapid clearance from the peritoneal cavity. In contrast, among the 8 rats treated by PDLLA-PEG-PDLLA hydrogel, only one rat obtained moderate adhesions between the omental and the sutured incision, the remaining animals did not suffer from any adhesions, and the defects were almost completely regenerated within 14 days (Fig. 7A(f)). Compared with other groups, the median adhesion scores of the PDLLA-PEG-PDLLA hydrogel-treated animals was significantly lower (p < 0.05, Mann-Whitney μ test). Simultaneously, the PDLLA-PEG-PDLLA hydrogel had disappeared completely from the injured sites and could not be observed in the parietal and visceral surfaces due to the degradation and absorption of the hydrogel.

A histological observation of the adhesion sites was also performed, as shown in Fig. 7B.Tissues taken from the control group and HA hydrogel group showed that the cecal muscular layer was fully fused to the abdominal wall musculature, with a large amount of intervening inflammatory cells and fibroblasts in the adhesion sites (Fig. 7B(a)). Conversely, the defects treated with PDLLA-PEG-PDLLA hydrogel had been re-epithelialized and showed integral neo-mesothelial cells layers on top of the abdominal or cecal muscle at the 14th day, which were similar to that in the normal tissues (Fig. 7B(b,c)). Overall, the PDLLA-PEG-PDLLA hydrogel showed satisfactory efficacy in post-operative peritoneal adhesion prevention in rats.

## Conclusion

We are reporting a novel reversible thermogelling PDLLA-PEG-PDLLA triblock copolymer, which underwent a sharp sol-gel transition upon heating. The gelation temperature could be adjusted in a physiologically important temperature range by modulating molecule weight, block length and polymer concentration. Aggregation of micelles was thought to be involved in the sol-gel transition and no crystallization formed during the gelation. The gelation was proved to be thermally reversible and the polymer solutions showed pronounced sol phase stability at room temperature. Thus, injection could be readily preformed without the risk of syringe clogging that was convenient to administer. Both the *in vitro* and *in vivo* investigations illustrated the acceptable biocompatibility and biodegradation of the novel physical hydrogel. Furthermore, the PDLLA-PEG-PDLLA hydrogel system was found to be highly effective in reducing the post-operative adhesion formation and very convenience in operation. Such an injectable, biocompatible, biodegradable and thermoreversible hydrogel could be considered as an attractive biomaterial for sustained drug delivery, tissue regeneration applications or other medical applications.

## Methods

### Materials and Animals

Poly(ethylene glycol) (PEG, *Mn* = 1500, 1000, 2000 respectively), stannous octoate (Sn(Oct)_2,_ 95%), ε-caprolactone (ε-CL), 3-(4,5-dimethylthiazol-2-yl)-2,5-diphenyl-tetrazolium bromide (MTT) were purchased from Sigma-Aldrich (USA). D,L-lactide (D,L-LA) was bought from Daigang chemicals, Jinan, China. Dulbecco’s modified Eagle’s medium (DMEM) was supplied by Gibco (Grand Island, NY, USA).Other chemical agents used in this work were bought from Kelong Chemical, Co., Ltd., Chengdu, China. They were all analytical pure grade and used as received.

Sprague-Dawley (SD) rats (female, 180 ± 20 g) and Balb/c mice (female, 20 ± 2 g) were purchased from the Experimental Animal Center of Sichuan University (Chengdu, China). The rats were housed in a specific pathogen-free (SPF) environment with a consistent temperature of 25 ± 2 °C and a relative humidity of 50–60% under 12 hrs-light-dark cycles. Free access to food and water was allowed. All animals would be in quarantine for at least a week before treatment. The animal experiments were approved by the Animal Care and Use Committee of Sichuan University (Chengdu, China) and were carried out in compliance with the approved guidelines (IACUC-S200904-P001).

### Synthesis of PDLLA-PEG-PDLLA copolymers

The triblock PDLLA-PEG-PDLLA copolymers were prepared by ring-opening copolymerization of D,L-lactide in the presence of PEG using stannous octoate as catalyst. A typical PDLLA-PEG-PDLLA copolymer with molecular weight of 4,500 Da (marked as L1500-E1500-L1500) was synthesized as follows: in brief, PEG (20.00 g, 13.33 mmol) was heated in vacuum at 100 °C for 1 h to eliminate the trace amount of water. After the flask was cooled to room temperature, D,L-lactide (40.00 g, 277.78 mmol) and Sn(Oct)_2_ (0.18 g, 0.44 mmol) were added. The reaction was carried out at 140 °C for 12 h under argon protection. Finally, the resulting PDLLA-PEG-PDLLA block copolymer was dissolved in ethanol (60 ml), and reprecipitated from the filtrate using excess pre-cold n-pentane (600 ml), the sediment was vacuum dried to constant weight at 45 °C. Other PDLLA-PEG-PDLLA copolymers with different molecular weight and blocks were synthesized similarly. In this paper, the copolymers were denoted as L_B_–E_A_–L_B_ (PLEL), where A and B represent the theroretical number average molecular weights (Mn) of PEG and PDLLA blocks respectively. For comparison, PCL-PEG-PCL (PCEC, 1000-1000-1000) triblock copolymers were prepared by ring-opening copolymerization of ε-CL initiated by PEG, which was reported previously by our group^26^. The entire list of the synthesis of all the copolymers can be found as Supplementary Table S1.

### Characterization of physicochemical properties

#### *Nuclear magnetic resonance (* ^*1*^ *H-NMR)and gel permeation chromatography (GPC)*

^1^H-NMR spectra (in CDCl_3_) was performed at room temperature with a Varian 400 spectrometer (Varian, USA) at 400 MHz to characterize chemical composition of the copolymers. The samples were dissolved in CDCl_3_, and the chemical shifts were given in ppm using tetramethylsilane (TMS) as an internal reference. GPC (Agilent 110 HPLC, USA) was also used to determine the macromolecular weight and macromolecular weight distribution of the prepared copolymers. The samples were dissolved in freshly distilled tetrahydrofuran (THF) at a concentration of 1 mg/ml. THF was eluted at a rate of 1.0 ml/min. The molecular weights of samples were calibrated with polystyrene (PS) as standard.

#### Characterization of micelle morphology and size

The self-assembled PDLLA-PEG-PDLLA copolymer micelles in water were characterized by transmission electronic microscopy (TEM) and dynamic light scattering (DLS) measurements. The morphology of micelles was observed under a transmission electron microscopy (TEM, H-6009IV, Hitachi, Japan). Before observation, the samples were prepared by placing a drop of micelles suspension (0.1 wt%, 20 °C) onto a copper grid covered with nitrocellulose. Then they were negatively stained with phosphotungsticacid and dried in air. Dynamic light scattering (Nano-ZS 90, Malvern, Worcestershire, UK) was used to determine the size distribution of the micelles at the copolymer concentrations of 1 wt% and 10 wt%. Measurements were performed at increasing temperatures from 4 °C to 45 °C and each temperature was kept for 10 min to equilibrium prior to measurement.

#### Phase diagram measurement

The sol (flow) - gel (no flow) phase transition temperatures of copolymers in water were determined using the test-tube-inversion method with a 4-mL vial test tube with an inner diameter of 10 mm at a temperature interval of 1 °C, from 0 °C to the temperature when precipitation occurred. The phase transition was observed visually by inverting the vials, and a gel was defined when no significant flow was observed within 1 min as described in reported papers^13^,^23^. The transition temperature is an average of three measurements for each point.

#### Dynamic rheological study of PDLLA-PEG-PDLLA solutions

Rheological measurements of the PDLLA-PEG-PDLLA copolymer solutions with determinate concentrations were carried out by using an HAAKE Rheostress 6000 rheometer (Thermo scientific, USA) using parallel plates. The cold samples were placed between parallel plates with diameter of 20 mm and with a gap of 1 mm, and carefully overlaid with a thin layer of low-viscosity silicone oil to minimize solvent evaporation. During temperature sweep experiments, the heating and cooling rates were 1 °C/min. The storage modulus (G’) and loss modulus (G”) were measured as functions of temperature. The data were collected under a controlled stress (4.0 dyn/cm^2^) and a frequency of 1.0 Hz. Gelation times of the copolymer solutions at 37 °C were also investigated, where the G’ and G” were recorded as functions of time. The gelation time was defined as the time when G’ became higher than G”. Change in G’ of PCL-PEG-PCL (PCEC) copolymer solution (20 wt%) under heating and cooling process was also been studied for comparision.

### Polarized optical microscopy

Polarized optical microscopy tests of the PDLLA-PEG-PDLLA (PLEL) copolymer solution (25 wt%) and PCL-PEG-PCL (PCEC) copolymer solution (20 wt%) were carried out by using a polarized optical microscope (Olympus; Bh-753pw) to study the morphology during the gelation. The polymer aqueous solution was placed between two slide glasses, and the microscopic image was photographed at 0 min and 1 hour at 20 °C. Then, the polarized optical microscopic images of the instantaneously formed gel by heating the slide at 37 °C were also performed.

### X-ray diffraction aAnalysis

Crystallographic assays were performed on the PDLLA-PEG-PDLLA (PLEL) copolymer hydrogel and PCL-PEG-PCL (PCEC) hydrogel by PHILIPS X-ray Diffraction (XRD, X’ Pert Pro, MPDDY 1291) using Cu KR radiation. Samples were scanned from 10^o^ to 60^o^ at a scanning speed of 1 ^o^ /min.

### *In Vitro* cytotoxicity tests

Murine L929 cells and human umbilical vein endothelial (HUVEC) cells (American Type Culture Collection, Rockville, MD) were chosen to assess the cell cytotoxicity of the synthesized polymer and hydroge by MTT assay. The cells were cultured in Dulbecco’s modified Eagle’s medium (DMEM, Gibco) containing 10% fetal bovine serum (FBS, Gibco, USA), supplemented with 50 U/ml penicillin and 50 U/ml streptomycin at 37 °C in 5% CO_2_. First, the prepared PDLLA-PEG-PDLLA hydrogel was extracted using DMEM with 10% FBS for 24 h. Then, sequential dilutions of the stock solution were carried out to obtain a series concentrations of the leachates. Cell suspensions were distributed in a 96-well plate at a density of 3 × 10^4 ^cells/well and incubated for 24 h. The medium and then was replaced with fresh medium with a different concentration of PDLLA-PEG-PDLLA copolymer or hydrogel leachates and incubated up to another 48 h. Subsequently, 20 μl of MTT (3-(4,5-dimethylthiazol-2-yl)-2,5-diphenyl-tetrazolium bromide, Sigma-Aldrich, 5 mg/ml) was added to each well and the cells were further incubation at 37 °C for another 4 h. The precipitated formazan was dissolved in 150 μl DMSO and the absorbance at 570 nm was measured using an ELISA microplate reader (Bio-Rad). The cytotoxicity was defined as the relative viability (%), with no block copolymer or hydrogel leachate in the culture media as 100%. All the data were expressed as the mean ± SD (n = 5).

### *In vitro* hemolysis assays

The hemolytic test was performed on solution of PDLLA-PEG-PDLLA (S3) copolymer in the normal saline solution *in vitro* as the reported method^3^,^33^. In this experiment, 2.5 ml samples with different concentrations were added to 2.5 ml of off-fiber rabbit erythrocyte suspension (2%) in normal saline and incubated at 37 °C °C. Normal saline and distilled water were employed as negative and positive control, respectively. After incubated at 37 °C for 3 h, the erythrocyte suspension was centrifuged at 2000 rpm for 10 min, and then color of the supernatant was compared. Absolute achromatic supernatant solution implies that there is no hemolysis. In contrast, red supernatant solution means hemolysis. Then the supernatant of the erythrocyte suspension were collected and detected on a UV/Vis spectrophotometer (Lambda 35, Perkin Elmer) at 540 nm to determine the hemolytic ratio. The hemolytic ratio was calculated according to the following equation:


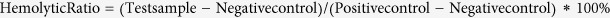


All results were estimated from the data of three independent experiments, and all data were expressed as the mean ± SD (n = 3).

#### *In vitro* degradation of hydrogel

*In vitro* degradation behavior of the hydrogel was measured by the method as the previous report under simulated physiological conditions^25^. Briefly, the polymeric aqueous solution (25 wt%, 1 ml) was injected into a test tube and incubated in a shaking bath at 37 °C with 50 strokes /min. After 10 min, 9 ml of PBS solution (pH 7.4) was added to the formed gels. The buffer solution was replaced by a fresh one every 4 day to maintain the medium pH. At pre-determined times, some samples were taken out of the shaking bath, the buffer was removed, and the remaining gels were freeze-dried until a constant weight. For analysis of the dried sample, experiments of ^1^H NMR and GPC were done. pH change of the medium was measured with a pH meter at designated time intervals before the medium was replaced by a fresh one.

### *In vivo* gel formation, degradation, and biocompatibilty tests

*In vivo* gel formation and degradation tests upon dorsal subcutaneous administration were performed in BALB/c mice. 0.5 ml aqueous solutions of PDLLA-PEG-PDLLA triblock copolymer (25 wt% in the PBS solution, pH 7.4) were dorsal subcutaneously injected into mice by a syringe with a 25-gauge needle at room temperature. At predetermined time, three mice were sacrificed by cervical dislocation. The injection sites were carefully opened, and then photographs of the remaining gels were taken. The remaining gels in the animals were taken out for analyses of MWs by GPC. Meanwhile, the muscles surrounding the subcutaneous implants and major organs including heart, liver, spleen, lung and kidney were surgically removed followed by hematoxylin-eosin (HE) staining for further histopathological examination.

### *In vivo* application and evaluation of adhesion-prevention effect in rat model

The anti-adhesion efficacy of the PDLLA-PEG-PDLLA hydrogel was tested using a Sprague-Dawley (SD) rat model of sidewall defect-cecum abrasion^31^,^37^. In this study, twenty-four SD rats were model animals and were randomly divided into three groups (n = 8). All of the model animals were treated humanely during the study.

In surgery, aseptic technique was applied throughout the experimental period. The rats were completely anesthetized by intraperitoneal injection of chloral hydrate (10%, 3 mL/kg), and placed in the supine position, shaved in the abdominal area, upon which the abdomen was exposed by a ventral midline incision. Abdominal adhesions were induced according to the method of Yoon Yeo. *et al*.^31^. Firstly, a 2 × 2 cm parietal peritoneal defect with punctuate hemorrhage was created using scalpel in the right lateral abdominal wall until the peritoneum as well as underlying partial muscular layer were excised from the abdominal wall. Secondly, a 2 cm^2 ^of the cecal serosa was abraded with sterile dry surgical gauze until oozing of blood was observed from the serosa but not perforated. Then the two injured surfaces were juxtaposed with 3-0 silk sutures in order to contact. For the treatment group, 1 ml of the PDLLA-PEG-PDLLA solution (25 wt% in the PBS solution, pH 7.4) was uniformly painted on the abdominal wall defect as well as the damaged cecum surface, respectively. The hydrogel was allowed to fully gelatinize (approximately 2 min). Then the incisions of all animals were closed in two layers with 3/0 surgical silk. For the positive control group, the defect was treated with 1 ml HA hydrogel (a commercialized anti-adhesion hyaluronic acid hydrogel, Xinkeling®). The remaining eight rats with untreated defects were served as negative controls. Two weeks after the surgery, the rats were sacrificed by cervical dislocation and the anti-adhesion efficacy was evaluated by two observers in a double-blinder manner. Each animal was evaluated according to the following standard adhesion scoring system, which has been widely used in this field: score 0 = no adhesion; score 1 = mild adhesion, easily separable intestinal adhesion; score 2 = moderate intestinal adhesion, separable by blunt dissection; score 3 = severe intestinal adhesion, adhesion requiring sharp dissection^38^. In addition, we took specimens from the damaged cecum, damaged abdominal wall, and adhesion-associated tissues. Then, the specimens obtained were fixed in 10% formalin, embedded in paraffin, sectioned and stained with HE staining for histological examinations.

### Statistical analysis

The statistical analysis was performed using SPSS 15.0 software (Chicago, IL, USA). The results are expressed as means ± SD. Since adhesion scores did not always follow a normal distribution, statistical inferences were made using Mann-Whitney μ tests. Statistical significance was determined as P ≤ 0.05.

## Additional Information

**How to cite this article**: Shi, K. *et al*. Synthesis, characterization, and application of reversible PDLLA-PEG-PDLLA copolymer thermogels *in vitro* and *in vivo*. *Sci. Rep*. **6**, 19077; doi: 10.1038/srep19077 (2016).

## Supplementary Material

Supplementary Information

## Figures and Tables

**Table 1:** The obtained PDLLA-PEG-PDLLA triblock copolymers in this study.

code	PDLLA-PEG-PDLLA	Total Mn^a^	Total Mn^b^	Total Mn^c^	PDI^c^	Solubility d	Sol-gel^e^
S1	L_1000_-E_1000_-L_1000_	3000	2993	2393	1.51	Soluble	Sol-gel
S2	L_1300_-E_1500_-L_1300_	4100	4153	3412	1.61	Soluble	Sol-gel
S3	L_1500-_E_1500_-L_1500_	4500	4495	4281	1.58	Soluble	Sol-gel
S4	L_1700_-E_1500_-L_1700_	4900	4851	4539	1.57	Insoluble	–
S5	L_2000_-E_2000_-L_2000_	6000	5922	5620	1.51	Insoluble	–
S6	L_1000_-E_2000_-L_1000_	4000	3907	3578	1.54	soluble	–

^a^Theroretical value, calculated according to the feed ratio.

^b^Calculated from ^1^H NMR results.

^c^Obtained by GPC tests with respect to PS standard.

^d^Solubility in water at 4 °C to 30 °C.

^e^Sol-gel phase transition of the copolymers aqueous solutions, investigated by test-tube-inversion method.

**Table 2:** Evaluation of peritoneal adhesions in the rat model.

Adhesions	Control group		HA hydrogel group		PLEL hydrogel group	
	(n = 8)		(n = 8)		(n = 8)	
	Frequency	Percentage	Frequency	Percentage	Frequency	Percentage
Score 0	0	0	0	0	7	87.5
Score 1	0	0	1	12.5	0	0
Score 2	0	0	3	37.5	1	12.5
Score 3	8	100	4	50	0	0
Median score	3(3–3)		3(3–3)		0(0–2)*	

Score 0 = no adhesion; score 1 = mild adhesion; score 2 = moderate intestinal adhesion;

score 3 = severe intestinal adhesion.

*Significantly different with the values of control group, P ≤ 0.05, Mann-Whitney μ tests.
