# Supplementary material for: Synthesis, characterization, and application of reversible PDLLA-PEG-PDLLA copolymer thermogels in vitro and in vivo
Source: Sci Rep. 2016 Jan 11;6:19077. doi: 10.1038/srep19077 (PMC4707506; doi:10.1038/srep19077)
Supplement: Supplementary Information [file srep19077-s1.pdf]

SUPPORTING INFORMATION

Synthesis, characterization, and application of reversible PDLLA-PEG-PDLLA copolymer thermogels *in vitro* and *in vivo*

Kun Shi , Ya-Li Wang, Ying Qu, Jin-Feng Liao, Bing-Yang Chu, Hua-Ping Zhang, Feng Luo, Zhi-Yong Qian *

State Key Laboratory of Biotherapy and Cancer Center, West China Hospital, Sichuan University, and Collaborative Innovation Center for Biotherapy, Chengdu, 610041, China

The file includes:

Figuer S1. ^{1}H-NMR spectrum of PDLLA-PEG-PDLLA copolymer.

Figure S2. H&E staining of major organs after dorsal subcutaneous administration of PDLLA-PEG-PDLLA hydrogel for 8 weeks (×200).

Table S1. The synthesis of copolymers in this study

* Qian ZY is the corresponding author for this work. Tel/Fax: +86-28-85501986, E-mail: anderson-qian@163.com and zhiyongqian@scu.edu.cn.

SUPPLEMENTARY FIGURES

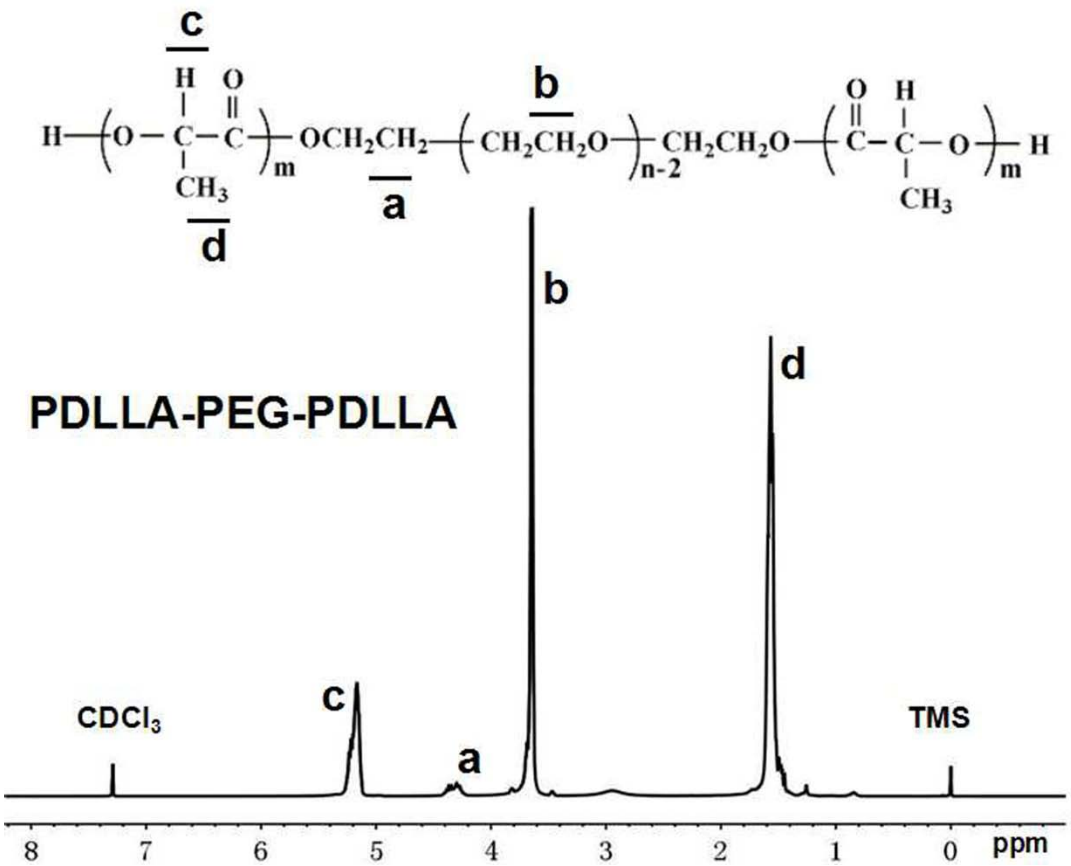

Figure S1. ^{1}H-NMR spectrum of PDLLA-PEG-PDLLA copolymer.

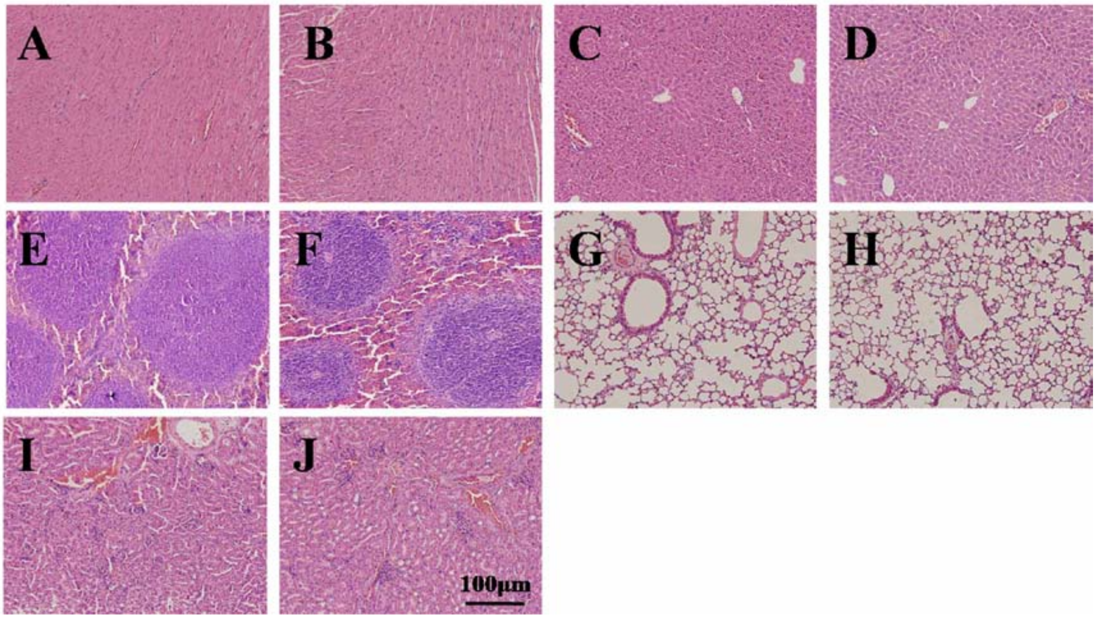

Figure S2. HE-staining of major organs after dorsal subcutaneous administration of PDLLA-PEG-PDLLA hydrogel for 8 weeks (×200). Mice heart, liver, spleen, lung and kidneys of control group (A, C, E, G, I) and hydrogel-treated group (B, D, F, H, J) respectively. The images was representative of n = 3.

SUPPLEMENTARY TABLES

Table S1: The synthesis of copolymers in this study

code	Copolymers	Feeding amounts			Solvent	Precipitant	Yield
		PEG (g)	D,L-lactide (g)	$Sn(Oct)_2$ (g)	ethanol (ml)	n-pentane (ml)	%
S1	L_{1000}-E_{1000}-L_{1000}	20.00	40.00	0.18	60	600	91.5
S2	L_{1300}-E_{1500}-L_{1300}	20.00	34.67	0.16	55	550	92.4
S3	L_{1500}-E_{1500}-L_{1500}	20.00	40.00	0.18	60	600	93.2
S4	L_{1700}-E_{1500}-L_{1700}	20.00	45.33	0.20	65	650	92.5
S5	L_{2000}-E_{2000}-L_{2000}	20.00	40.00	0.18	60	600	91.6
S6	L_{1000}-E_{2000}-L_{1000}	20.00	20.00	0.12	40	400	93.7
PCEC	C_{1000}-E_{1000}-C_{1000}	20.00	40.00	0.18	60	600	90.8
